# *Oregano* Extract Added into the Diet of Dairy Heifers Changes Feeding Behavior and Concentrate Intake

**DOI:** 10.1155/2016/8917817

**Published:** 2016-12-27

**Authors:** Giovani Jacob Kolling, Dejani Maíra Panazzolo, Alexandre Mossate Gabbi, Marcelo Tempel Stumpf, Marcel Batista dos Passos, Eduardo Augusto da Cruz, Vivian Fischer

**Affiliations:** ^1^Animal Science Department, Federal University of Rio Grande do Sul, Bento Gonçalves Avenue 7712, District Agronomy, 91540-000 Porto Alegre, RS, Brazil; ^2^School of Agroecology, Federal University of Rio Grande (FURG), Marechal Floriano Peixoto Avenue 2236, 96170-000 São Lourenço do Sul, RS, Brazil; ^3^Veterinary Large Animal Hospital, University of Brasilia, Granja do Torto, 70636-200 Brasília, DF, Brazil

## Abstract

This experiment aimed to describe the effects of* Oregano* extract (OE) inclusion into the concentrate fed to dairy heifers on physiological parameters, feeding behavior, intake, and performance. Thirty-two Holstein heifers were randomly distributed into four treatments: C = control, without addition of OE; OE2.5 = 2.5 g; OE5.0 = 5.0 g and OE7.5 = 7.5 g of* Oregano* extract per heifer/day. Feeding behavior and concentrate intake were assessed individually every day and total dry matter intake (DMI) was determined on the last week of the trial. Compared to control group, OE7.5 reduced by 32% the latency time to approach the feed bunk but increased by 6% the time spent eating the concentrate. Each inclusion of 2.5 grams of OE into the concentrate increased the occurrence of postingestive licking the feed bunk with abundant saliva production 1.2 times (*P* < 0.01) and tended to increase the occurrence of sneeze events 1.2 times (*P* < 0.10). No statistical difference was detected between treatments for total DMI, but concentrate DMI was 9% lower for OE7.5 when compared to control and OE2.5. The inclusion of 7.5 grams/day of OE causes small but negative effects in feeding behavior and concentrate intake, without change on total dry matter intake.

## 1. Introduction

Rearing replacement young dairy animals under intensive dairy system is expensive, requires extensive labor [1], and usually requires the anticipation of age at first calving without impairing body development and future milk production [2]. Practices to optimize this process may include the use of phytochemicals, which can improve aspects related to animal health, rumen efficiency, and weight gain. In order to increase food security and reduce negative environmental impacts, there is an increasing demand for the use of natural compounds in animal production systems. Essential oils, characterized as aromatic compounds with high volatility, are phytochemicals that can be extracted from several parts of plants, that is, flowers, leaves, and seeds (for a revision in this subjected, see Can Baser and Buchbauer [3]) and have the potential to alter animal growth. Since essential oils are natural products, they are being accepted by several segments of society and are considered an alternative to antimicrobials. Studies based on the supply of essential oils for ruminants, such as* Oregano* derivatives, were primarily based on assessing their effects on rumen metabolism [4–7], with few studies addressing their effects on behavior, with controversial results on intake and performance.

The use of* Oregano* (*Origanum vulgare*) extract can modify the rumen metabolism due to its antimethanogenic and defaunation actions [8]. Bampidis et al. [9] did not find positive effects of feeding dried* Oregano* leaves to growing lambs on intake, feed conversion, weight gain, body development, oxidative stress, and meat quality. Supplementation with distinct blends containing* Oregano* for dairy calves did not affect intake and body weight gain [10] but reduced dry matter intake (DMI) of cows in early lactation without affecting milk production [11]. Many factors may determine the variability of the published results: complex gastrointestinal tract of ruminants, impact of the basal diet on the relationship between rumen microbiota; intrinsic variability of plants extracts; extrinsic conditions that may affect concentration and bioavailability of phytochemicals such as weather conditions, harvest season, dose, extraction method, and presentation form [6].

The main essential oil contained in* Oregano* is carvacrol, followed by thymol [12]. It is important to emphasize that volatile compounds in the* Oregano* extracts may exert negative effects on intake, especially when high doses are used [6, 13]. In addition to changes in rumen microbiota, secondary compounds of plants, especially essential oils, may influence the neuronal activity through modulation of neurotransmitters as dopamine and serotonin, which potentially modify animal's response to the environment [14, 15]. Since essential oils alter rumen's functioning characteristics and neuronal activity, the potential effects on animals' behavior should be considered.

Despite these results, scientific studies assessing the influence of essential oils on ruminants' behavioral parameters, responses to physiological aspects, and performance are still insufficient. We hypothesized that* Oregano* extract may affect feeding behavior and feed intake and, in consequence, animal performance. In this sense, this study aimed to evaluate physiological parameters, feeding behavior, dry matter intake, and performance of dairy heifers receiving* Oregano* extract in the diet.

## 2. Material and Methods

### 2.1. Local Description, Animals, and Management

The experimental procedures followed the Brazilian Federal Constitution on scientific use of animals and were approved by the Ethics Committee on Animal Use of the Federal University of Rio Grande do Sul, protocol number 18510. The experiment was conducted in a commercial dairy farm (27°88′S 54°18′W 372 meters) in Rio Grande do Sul State, Brazil, from April to June 2014, during the autumn season. Temperature and relative humidity ranged from 6.8 to 27.7°C and from 43 to 98%, respectively. Values of temperature and humidity were used to calculate the temperature-humidity index (THI; equation by Johnson et al. [16]). During the trial THI varied between 55.2 and 72.3 and precipitation was almost null.

The trial lasted for 77 days and it was divided into two periods: a preexperimental period of 21 days (day −21 until day 0), in which animals were adapted to the new diet (with the exception of* Oregano* extract) and management conditions, and a main experimental period of 56 days (day 1 until day 56). Thirty-two Holstein heifers were used. They were, on average, 18.4 ± 4.2 months old and presented 424.2 ± 94.4 kg of body weight (BW). The animals were randomly distributed into four treatments: C = control, without addition of OE; OE2.5, OE5.0, and OE7.5, which received a daily dose of 2.5, 5.0, and 7.5 grams of* Oregano* extract (OE) mixed in the concentrate, respectively. The* Oregano* extract was supplied in a powder form [Orego-Stim®, Meriden Animal Health Ldta. (Northampton, UK) redistributed in Brazil by Advet Animal Nutrition] containing 5%* Oregano* essential oils of* Origanum vulgare *subsp.* hirtum* plants and 95% of inert carrier. The main essential oils of this product are carvacrol (80–82%), thymol (2.5 to 3%), p-cymene (3.5 to 9%), and Y-terpinene (2.0 to 5.5%). Therefore, the commercial product provided the following amount of carvacrol: OE2.5 = 101.25, OE5.0 = 202.5, and OE7.5 = 303.75 mg of carvacrol/heifer; when expressed as mg per kg of BW the amount of carvacrol was OE2.5 = 0.202, OE5.0 = 0.438, and OE7.5 = 0.605.

Heifers were brought from pasture to the barn every day at 07:00 h and were individually restrained at the feed bunk, where they received 2.5 kg of concentrate from 07:30 h to 09:30 h (GMT −03:00).* Oregano* extract was mixed into the concentrate immediately before feeding and the refusal was removed every day prior to the feeding of 16 kg of maize silage and 2 kg of grass hay, which occurred from 10:30 h to 11:30 h. At 11:30 h heifers were released from the feed bunk and, until 07:00 h of next day, they had free access to a paddock with* Cynodon dactylon *pasture.

### 2.2. Diet Composition

The diet was formulated to allow an average daily gain of 0.7 kg/day ([17] NRC, 2001). The concentrate was composed of soybean meal, wheat middling, soybean hulls, ground corn, mineral supplement Avant Milk, and mycotoxin adsorbents in proportions of 360, 250, 200, 130, 50, and 10 g per kg, respectively. Each kilogram of multicore Avant Milk contained monensin (1.000 mg), calcium (215 to 240 mg), and minimal concentrations of phosphorus (50 mg), sodium (90 mg), magnesium (15 mg), sulfur (15 mg), iron (1.000 mg), zinc (2.600 mg), copper (600 mg), manganese (880 mg), iodine (32 mg), cobalt (12 mg), selenium (16 g), and vitamins A, D3, and E.

Samples of silage, hay, and concentrate were collected once a week. Pasture samples were collected by hand-plucking every week near the place where heifers were grazing. The contents of NDF [18], dry matter, crude protein [19], IVDDM, and IVDOM [20] were determined, and results are showed in Table 1.

### 2.3. Measurements

All assessments were performed in the morning, after feeding concentrate in feed bunk. On days −6, 9, 23, 37, and 51, urine samples were obtained following spontaneous release or after a vulvar massage. Immediately after sampling, urinary pH was measured with Microprocessor Bench Q400MT. Physiological measures were taken on days −7, 2, 16, 30, and 44. Rectal temperature (RT) was obtained with the use of a veterinary clinical thermometer inserted next to the animals' rectum wall at a depth of approximately 3.5 cm during three minutes. Respiratory rate (RR) and heart rates (HR) were assessed through stethoscope auscultation during 30 seconds (then multiplied by two). On days −8, 7, 21, 35, and 49 heifers were weighed to calculate the average daily gain (ADG) and the body condition score (BCS) was assessed: 1: extremely lean and 5: extremely fat [21].

The animals were visually and continuously observed by two trained observers, from 07:30 h to 09:30 h (concentrate supply period) during all experiment period. Behavioral activities registered were latency to visit the feed bunk, latency time to start eating the concentrate, time spent eating the concentrate, number of events of vocalizations of high and low magnitude, sniffing, coughing, sneezing, interruptions in the intake concentrate for more than one minute, tongue rolling, and licking the feed bunk. These behaviors are described in the ethogram of Table 2. From 10:30 h to 11:30 h heifers were visually observed at a 10 min interval for time spent eating silage plus hay.

Concentrate intake was calculated daily as the difference between the amount of concentrate offered and the amount left in the feed bunk. Before feeding silage and hay, the concentrate refusal was removed and weighed. Total DMI was estimated indirectly. Fecal production was estimated with chromium oxide. Each heifer received 10 g/day of chromium oxide mixed with concentrate for 10 days (from day 32 until day 41) with posterior feces collection on the last 5 days (from day 38 until day 42). The fecal production was calculated dividing chromium concentration (mg/kg) corrected by the mean recovery rate of 90.6% [22] in the feces by the amount supplied in the feed. DMI was calculated by dividing the fecal production by (1–*in vitro* digestibility of dry matter of the diet) [20, 23].

### 2.4. Experimental Design and Statistical Analysis

The experimental design was completely randomized with repeated measurements, with heifers as experimental units. Continuous data about physiological parameters (urine pH, rectal temperature, respiratory rate, and heart rate), performance (ADG and BCS), and time spent in feeding behaviors and concentrate intake were submitted to analysis of variance. The effects of treatment, day, and interaction between treatment and day were assessed using the procedure MIXED of the statistical software SAS Enterprise Guide 5.1 using the following parameters: method = REML, covariance matrix = CS, and repeated = day. Means were compared through LSMEANS option PDIFF. The total DMI was measured on the last week of the trial and it was submitted to variance analysis considering the effect of treatment. Traits measured on day 0 (the day before the first feeding with* Oregano* extract) were used as covariates. Linear and quadratic regression analyses were also performed using the PROC REG and testing the effect of dose of* Oregano* extract.

The number of behavioral events such as vocalizations of high and low magnitude, sniffing, coughing, sneezing, interruptions in the intake concentrate for more than one minute, tongue rolling, and licking the feed bunk was transformed into a binary response (0 = absence and 1 = presence) and further submitted to logistic analysis to calculate the likelihood of occurrence of the behavior using the procedure logistic of the statistical software SAS Enterprise Guide 5.1. The significance criterion was taken as *P* < 0.05 and tendency was taken as *P* < 0.10.

## 3. Results

Only the results for the effect of* Oregano* extract doses (treatment) will be presented, since there were no significant interactions between treatment and evaluation days. As linear and quadratic regression equations were not significant (*P* > 0.10) for all attributes, they will not be presented. The inclusion of* Oregano* extract did not affect the physiological parameters such as urine pH, rectal temperature, and respiratory rate (*P* > 0.05) but changed the heart rate (*P* < 0.05). Animals in OE5.0 group showed the highest heart rate. Heifers in OE7.5 group had lower concentrate intake than heifers in control and OE2.5 groups (*P* < 0.05). Total DMI, BCS, and ADG did not differ (*P* > 0.05) between treatments (Table 3).

Inclusion of each 2.5 g of OE into the concentrate increased the likelihood of postingestive licking the feed trough 1.2 times (*P* < 0.01) and tended to increase 1.2 times the odds of occurrence of sneeze events (*P* < 0.10). On the other hand, the inclusion of OE into the concentrate did not change the odds of occurrence of high and low magnitude vocalizations, coughing, sniffing, interruptions in the ingestion of the concentrate, and tongue rolling.

Latency time to visit the feed bunk was 32% lower for OE7.5 heifers than for the other groups (*P* < 0.05). In all groups there was no latency time to start eating the concentrate. Time spent eating the concentrate was increased in 6% for OE7.5 heifers (*P* < 0.05), with heifers in OE5.0 group presenting the lowest value. Inclusion of OE into the concentrate did not influence time spent eating silage plus hay, when heifers were restrained at the feed bunk (Table 4).

## 4. Discussion

The inclusion of spices, especially those containing essential oils, into the diet of ruminants is increasing due to their effects on ruminal fermentation [5, 6] and animal health [10, 24, 25], although there are reduced evidences of spices' effects improving animal performance [26]. In nonruminants, essential oils have been used in a wide scale due to their potential as replacement of antibiotics [27, 28]. When considering that ruminants are sensitive to the taste and smell of food [29], it is possible to hypothesize that using plant extracts with essential oils may elicit changes in ingestive behavior. However, to our knowledge, few studies evaluating the behavioral response to* Oregano* extract were conducted. The hypothesis that* Oregano* extract modifies the feeding behavior and intake of food was confirmed. Since food acceptance impacts intake, it is important to evaluate the effect of new additives added into the feed.

The significant negative effect of the highest dose (OE7.5) on concentrate DMI was probably due to the concentrate's flavor, as heifers decreased intake and extended the time to eat the concentrate. This result is in agreement with the findings of Launchbaugh and Provenza [13] who reported that lambs consumed less concentrate with strong than with light* Oregano* flavor and with Polat and El Sabry [30] who noticed extended ingestion time for sheep fed with* Oregano* essential oil. The high volatility of essential oils causes the strong smell and flavor, which could reduce feed palatability and animal feed intake [31, 32]. Usually, secondary plant compounds are defense mechanisms against predators, and some plants produce compounds with certain strong flavors to prevent grazing [13].

In the present study, with the range of doses of* Oregano* extract used (from 0.202 to 0.605 mg of carvacrol/heifer/day), an extreme strong or pungent odor of carvacrol was not perceived. Although animals in OE7.5 presented an extended time to approach the feed bunk, all heifers consumed concentrate readily, latency time to start eating the concentrated was virtually zero, which might indicate the absence of negative effects of carvacrol on animals' appetite. However,* Oregano* contains secondary compounds and essential oils that may depress palatability, possibly through aversive taste or postingestive feedback, what might explain the extended time to eat the concentrate.

According to Platel and Srinivasan [33], essential oils contained in spices are well recognized to stimulate gastric salivary flow and gastric juice production due to the stimulation of the nerve centers by the sense of smell and taste. The increase of postingestive licking and sneezing following elevation in OE levels might be due to the volatile compounds present in the* Oregano* extract. Carvacrol is able to cross the blood brain barrier and act on the central nervous system [14], affecting neurotransmitters as serotonin and dopamine in the brain [15] and thus the appetite [34].

Measurements of physiological parameters were performed to verify the health conditions and thermal comfort of the heifers, since they might modify their behavior on stressful conditions. Urine pH, RT, RR, and HR values were within normal range [35, 36] and indicate that heifers were healthy and unstressed. The variation in HR values, although statistically significant, was of small magnitude, within the physiological range and did not present a consistent dose-dependent variation.

The absence of statistical differences in ADG and BCS between groups was probably due to similar total DMI, nutrient intake, and the fact that these animals were not submitted to challenges, as they were not suffering from heat stress, nutritional restrictions or pathogens infections. The difference in concentrate intake was statistically significant, but as the magnitude was small, approximately 10%, it was not capable of affecting ADG and BCS. The absence of effect on DMI, ADG, and BCS is consistent with the results reported by Bampidis et al. [9] working with growing lambs and using comparable doses of dried* Oregano* leaves. On the other hand, Hristov et al. [5] fed* Oregano* leaves to dairy cows and observed a linear decrease in DMI and increased feed efficiency with* Oregano* supplementation.

## 5. Conclusion

*Oregano* extract has a characteristic taste and smell and when used as a spice for dairy heifers it may alter feeding behavior and intake. The inclusion of* Oregano *extract in dairy heifers concentrate does not change physiological parameters. Despite its characteristic of taste and flavor, feeding* Oregano* extract at 375 mg/heifer shows small depressive effects on the feeding time and concentrate intake of dairy heifers, without changing body weight gain and body condition score.* Oregano* extract does not expressively impair behavior, intake, and performance, encouraging further studies to verify its effects on immune system and metabolism.

## Figures and Tables

**Table 1:** Chemical composition of the experimental feeds (values are expressed as % of DM).

Items^1^	Concentrate	Silage	Hay	Pasture
NDF	30.04	45.99	75.52	61.14
CP	25.48	8.91	5.73	20.24
OM	90.75	96.47	93.67	89.56
IVDDM	88.19	69.37	56.13	71.95
IVDOM	90.33	70.22	55.33	72.56

^1^NDF = neutral detergent fiber; CP = crude protein; OM = organic matter; IVDDM = *in vitro* digestibility of dry matter; IVDOM = *in vitro* disappearance of organic matter g/100 g of DM.

**Table 2:** Ethogram with the description of behavioral activities performed by dairy heifers when fed with *Oregano* extract at the feed bunk.

Behavioral traits	Brief description
Latency to visit the feed bunk	Time elapsed since heifers entered the stall in the morning until they put the head in the restrainer (minutes)
Latency to eat the concentrate or silage plus hay	Time elapsed since heifers put the head in the restrainer until they start to eat the concentrate or silage plus hay (minutes)
Time eating the concentrate or silage plus hay	Effective time spent eating the concentrate or silage plus hay without pauses (minutes)
Vocalizations of low magnitude	Number of vocalizations performed with the mouth almost closed during their restrained period at the feed bunk
Vocalizations of high magnitude	Number of vocalizations performed with the mouth wide open during their restrained period at the feed bunk
Sniffing, coughing, sneezing	Number of events when heifer sniffed or coughed or sneezed during their restrained period at the feed bunk
Pauses	Number of times heifers interrupted eating the concentrate during one minute or more during their restrained period at the feed bunk
Tongue rolling	Number of events when heifers rolled their tongue during their restrained period at the feed bunk
Licking the feed bunk	If the feed bunk was licked with abundant production of saliva after concentrate consumption (0 = no, 1 = yes)

**Table 3:** Mean physiological measurements, performance, and dry matter intake of heifers receiving different doses of oregano extract with corresponding significance levels.

Items	Treatments (g of *Oregano* extract/heifer/day)					SEM
	0	2.5	5.0	7.5	*P* < *F*	
Urine pH	8.3	8.0	8.3	8.2	0.47	0.1
RT (°C)	38.6	38.6	38.6	38.6	0.64	0.0
RR (breaths/min)	46.4	45.0	45.7	46.4	0.54	1.0
HR (beats/min)	73.8^cb^	75.8^b^	79.0^a^	73.2^c^	<0.01	2.4
Concentrate DMI (kg/day)	2.2^a^	2.2^a^	2.1^ab^	2.0^b^	<0.01	0.0
Total DMI (% of BW)	2.8	2.7	3.3	2.5	0.18	0.7
Total DMI (kg/day)	13.5	13.4	14.7	12.2	0.18	2.0
Average daily gain (kg)	0.64	0.70	0.65	0.75	0.84	0.1
BCS (1–5)	3.9	3.9	3.9	3.8	0.63	0.0
BW (kg)	499	502	462	502	0.80	11.9

HR = heart rate; RR = respiratory rate; RT = rectal temperature; DMI = dry matter intake; BCS = body condition score; BW = body weight.

SEM = standard error of the mean.

^a, b, c^Values within a row with different superscripts differ significantly at *P* < 0.10.

**Table 4:** Treatments significance level (*T*), treatment × day interaction (TXD), and means of feeding behavior in the feed bunk.

Behavioral variables	Treatments (g of *Oregano* extract/heifer/day)				*T* ^1^	TXD^2^	SEM
	0	2.5	5.0	7.5			
Latency to approach the fed bunk (min)	3.7^a^	3.5^a^	3.6^a^	2.5^b^	<0.01	1.0	0.2
Latency to eat concentrate (min)	0	0	0	0	0.35	0.25	0
Time eating concentrate (min)	20.4^b^	20.2^b^	19.7^c^	21.6^a^	<0.0001	1.00	0.2
Time eating silage and hay (min)	63.1	60.5	62.1	61.3	0.60	0.9	2.0

^1^Effect of treatment.

^2^Effect of the interaction treatment × day.

SEM = standard error of the mean.

^a, b, c^Values within a row with different superscripts differ significantly at *P* < 0.05.
